# Supplementary material for: Full genome sequence analysis of African swine fever virus isolates from Cameroon
Source: PLoS One. 2024 Mar 21;19(3):e0293049. doi: 10.1371/journal.pone.0293049 (PMC10956809; doi:10.1371/journal.pone.0293049)
Supplement: S1 Fig — Raw reads from Illumina and Nanopore sequencing runs were mapped back against the assembled genomes of CAM1982 (A), CAM1994/1 (B) and CAM2018/lab1 (C) using Geneious Prime. Plots were displayed using the Integrative Genome Viewer. (PDF) [file pone.0293049.s001.pdf]

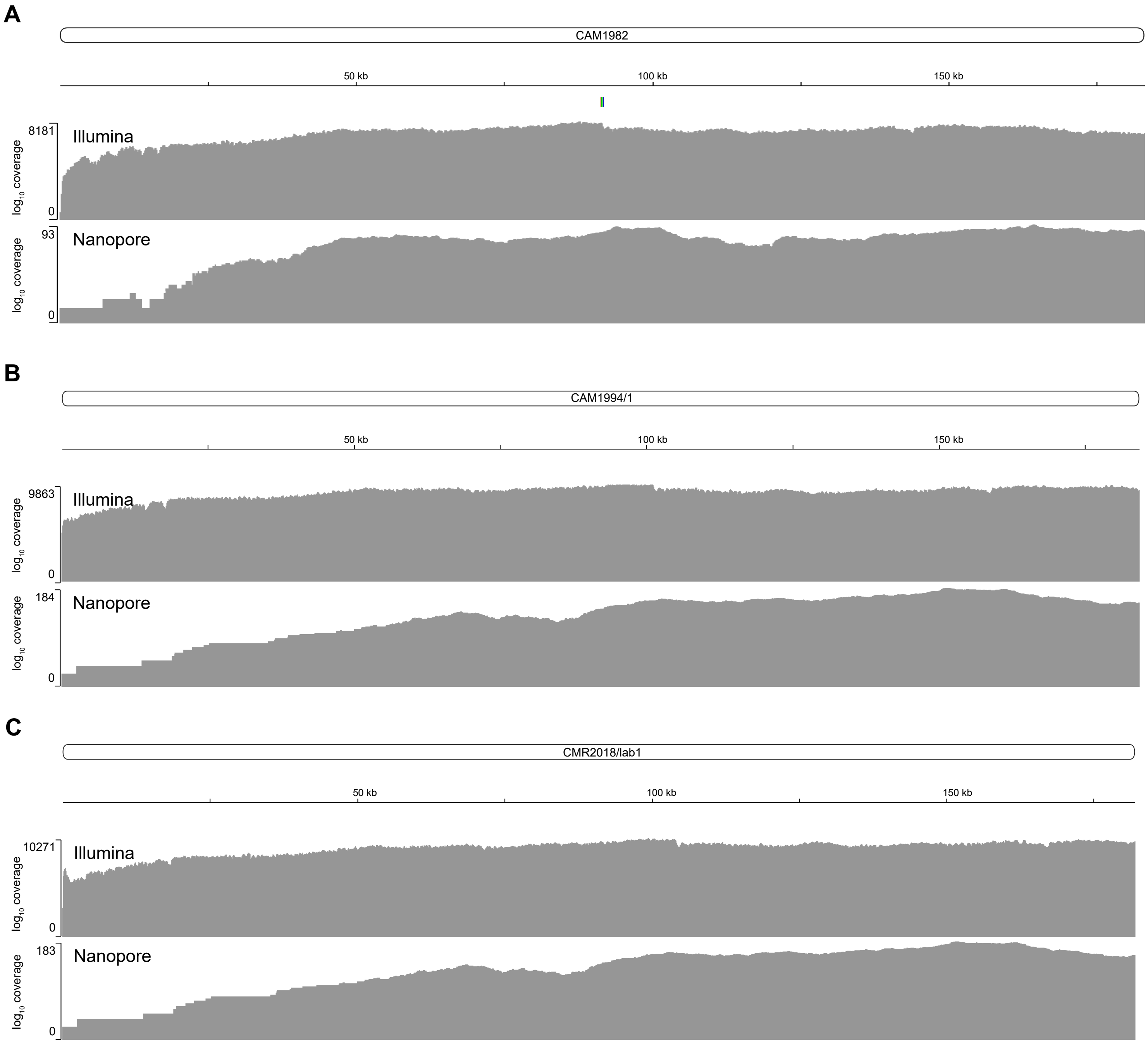

Supplemental Figure S1: Coverage plots. Raw reads from Illumina and Nanopore sequencing runs were mapped back against the assembled genomes of CAM1982 (A), CAM1994/1 (B) and CMR2018/lab1 (C) using Geneious Prime. Plots were displayed using the Integrative Genome Viewer.
