# Supplementary material for: Full genome sequence analysis of African swine fever virus isolates from Cameroon
Source: PLoS One. 2024 Mar 21;19(3):e0293049. doi: 10.1371/journal.pone.0293049 (PMC10956809; doi:10.1371/journal.pone.0293049)
Supplement: S1 Table — The positions of substitutions (>), insertions (^) and deletions (˅) relative to the Benin 1997/1 sequence (AM712239) are indicated along with the gene within which the substitution is found, as well as any potential functional consequences. (PDF) [file pone.0293049.s002.pdf]

Position in Benin (AM712239) Genome	CAM1982	CAM1994/1	CMR2018/lab1	Gene	Consequence
902			G>A	MGF360-1L	P145L
1363			vA	Intergenic homopolymer	
1461			vC	MGF360-2L	Frame shift and truncation. RYKKLCPKIIRWARFII* > RYKNCVRK*
3392	T>C		T>C	L83L	K63R
4690			T>C	MGF360-3L	Q59R
6798	^CC	^CC	^CC	MGF110-11L	Homopolymer. N-terminal change. MKYSWKNGGG > MEKWGGG
7671			vA	MGF110-12L MGF110-13L intergenic, homopolymer	
8187			^CC	MGF110-13L	Homopolymer. N-terminal change. MGGGDH > MKYSCKHGGGGDH
				MGF110-14L	C terminal truncation TWGGVTINNYL* > TWGGG*
9912			G>A	Intergenic	
10113	vG	^G	vG	Intergenic homopolymer	
10332		^G	^G	Intergenic homopolymer	
12086		^G	^G	Intergenic	
12329	^G	^GG		Intergenic homopolymer	
12339			G>A	Intergenic homopolymer	
12519	^GGG		^GGG	Intergenic homopolymer	
14142	^GG	^G	^G	Intergenic homopolymer	
14367	vGGG	vGGG	vGGGG	Intergenic homopolymer	
14359			C>T	Intergenic	
14495	^GG	^GG	^G	Intergenic homopolymer	
14695	^G	^GG		Intergenic homopolymer	
14873	C>T	C>T	C>T	J64R	Silent
15735			G>C	MGF300-2R	Silent

Position in Benin (AM712239) Genome	CAM1982	CAM1994/1	CMR2018/lab1	Gene	Consequence
17207			T>C	Intergenic	
18286		^A		Intergenic homopolymer	
19549	vATGTTATAACC			Intergenic repeat (two in Benin, CAM94 and CAM2018)	
24053	T>G		T>G	MGF360-12L	M260L
26588	G>A			MGF360-14L	H187Y
28139			G>A	MGF505-2R	R263K
29527			T>A	MGF505-3R	L170Q
34755	A>G		A>G	MGF505-6R	N497S
38957	C>T		C>T	MGF505-10R	H83Y
39980			C>T	MGF505-10R	L424F
42843	A>G			A151R	K7E
43593			^T	Intergenic homopolymer	
45261	T>C		T>C	A238L	Silent
46995			G>A	A859L	Silent
48371		A>G		A179L	I55T
48639			C>T	Intergenic	
49751	C>T		C>T	F317L	V143I
50194			^A	Intergenic homopolymer	
54233	T>C		T>C	F1055L	Silent
61227	T>C	T>C	T>C	EP1242L	K1073E
63280	A>G	A>G	A>G	EP1242L	Silent
69507			G>A	EP364R	Silent
70381			G>A	M1249L	Silent
73645			C>G	M1249L	D49E
75103			C>T	M448R	H425Y

Position in Benin (AM712239) Genome	CAM1982	CAM1994/1	CMR2018/lab1	Gene	Consequence
76117			^TGCACAAGTGCTTGCACAAGTGCTTGCACAAGTGCC	C44L	12 aa insertion ASTCASTCASTC
		^TGCACAAGTGCT		C44L	4 aa insertion ASTC
78864			G>A	Intergenic	
80990			C>T	C475L	D146N
81787			G>A	C315R	V104I
82421			^TTTAAACTAAACG	C315R	Duplication of repetitive sequence, no change to AA sequence
83113			A>C	C62L	S58A
84217	A>G		A>G	C962R	Silent
88802			C>T	B962L	R160H
91936	vGCTTTGGACCGGCCG	vGCTTTGGACCGGCCG	vGCTTTGGACCGGCCG	B169L	Deletion of repetitive sequence. Truncation, removes one copy of three PAGPK repeats
95363			G>A	B602L	Silent
96065				B602L	CVR, see Table X
96346					
96958			^G	Intergenic	

101764	vTGCGTATA CTGCCATTG CGTATACTG CCATTGCGT ATACTGCCA CTGCGTATG CTGCCAC TGCGTATAC TGCCATTGC GTATACTGC CATTGCGTA TACTGCCAC TGCGTATGC TGCCAC			B407L	Deletion of repetitive sequence, deletion of two of three copies of NGSIR repeat and one of two copies of SGSIR repeat
101765			vGCGTATACTG CCATT	B407L	Deletion of repetitive sequence, deletion of one of three copies of NGSIR repeat
107313	T>C		T>C	G1340L	T159A
107316			C>A	G1340L	V158L
109174			C>T	G1211R	Y445
118226			G>A	CP2475L	A381V
118366			G>A	CP2475L	Silent
121061	A>G	A>G	A>G	CP530R	I326V
130925			C>T	NP868R	Silent
140155			C>T	D345L	G189S
142963	T>C	T>C	T>C	P1192R	Silent
145605	G>A	G>A	G>A	P1192R	H1140R
151564	^G		^G	Intergenic homopolymer	
160666	T>C		T>C	E199L	E127G
160734	T>A*		T>A	E199L	Q104H *E199L SNP2
160793	C>G*			E199L	A85P *E199L SNP1
163962				E111R	Silent
164095			G>C	Intergenic	
164257			C>T	I267L	S258N
164364	A>G		A>G	I267L	Silent

Position in Benin (AM712239) Genome	CAM1982	CAM1994/1	CMR2018/lab1	Gene	Consequence
165718	T>C	T>C	T>C	I226R	Y180H
166580	A>G		A>G	I243L	Y4H
167573	T>C		T>C	I329L	N183S
168505			^TCTTCACATTCA	I215L	Duplication of repetitive sequence, additional DECE
169698			vA	I196L	Homopolymer. Truncation at C-terminus, LNLANILNTILCIILIKNV
170250			vA	Intergenic homopolymer	
170745	T>C	T>C	T>C	DP238L	K99R
171476			^GGG	MGF360-16R	Extra G at position 88
171997			^G	MGF360-16R	48 C-terminal truncation.
172176	vCC		^C	Intergenic homopolymer	
175204			G>A	Intergenic	
175737	^A		^A	Intergenic homopolymer	
176084			T>A	I8L	E19D
177456			C>T	Intergenic	
177708	^T		^T	Intergenic homopolymer	
177718			vA	Intergenic homopolymer	
177827		vC		Intergenic homopolymer	
178121			^C	MGF360-18R	Frame shift, 59aa deletion. Additional 10 aa changed due to new start codon. Coverage 351. <10% 5Cs
178290	A>G		A>G	MGF360-18R	K126E
178869	vCCC		vCCC	DP71L	Homopolymer, G4Δ
179486			G>A	Intergenic	
179544			C>T	Intergenic	
181868	vA	vA	vA	DP60R	Reconstitutes DP60R which is absent in AM712239
181867	A>C		A>C	DP60R	K8Q

Supplementary Table S1: Differences between CAM1982, CAM1994/1 and CMR2018/lab1 genomes and the Benin 1997/1 reference. The positions of substitutions (>), insertions (^) and deletions (v) relative to the Benin 1997/1 sequence (AM712239) are indicated along with the gene within which the substitution is found, as well as any potential functional consequences.
