# Supplementary material for: Full genome sequence analysis of African swine fever virus isolates from Cameroon
Source: PLoS One. 2024 Mar 21;19(3):e0293049. doi: 10.1371/journal.pone.0293049 (PMC10956809; doi:10.1371/journal.pone.0293049)
Supplement: Attachment — Submitted filename: Response to Reviewers.docx [file pone.0293049.s003.docx]

# Rebuttal

Thank you for the time taken to review our paper. I accept all of the reviewers comments and have changed the manuscript accordingly.

The missing abbreviations have been added to the abstract.

The first part of the results and discussion was intended as a preamble, but appreciate that as written it was very methodical. We have shortened this to “Samples from the three isolates were subject to both nanopore and Illumina sequencing.”.
